# Case report: *Candida krusei* spondylitis in an immunocompromised patient

**DOI:** 10.1186/s12879-020-05451-3

**Published:** 2020-10-08

**Authors:** Audrey J. C. Overgaauw, David C. de Leeuw, Susanne P. Stoof, Karin van Dijk, Joost C. J. Bot, Eef J. Hendriks

**Affiliations:** 1 Department of Internal Medicine, Amsterdam University Medical Center, location VUmc, Amsterdam, The Netherlands; 2 Department of Hematology, Cancer Center Amsterdam (CCA), Amsterdam University Medical Center, location VUmc, Amsterdam, The Netherlands; 3 Department of Medical Microbiology, Amsterdam University Medical Center, location VUmc, Amsterdam, The Netherlands; 4 Department of Radiology & Nuclear Medicine, Amsterdam University Medical Center, location VUmc, Amsterdam, The Netherlands

**Keywords:** Candidemia, *Candida krusei*, Spondylitis

## Abstract

**Background:**

Invasive infections with *Candida krusei* are uncommon and rarely complicated by spondylitis. Previous described cases were solely treated with antimycotic therapy, despite guidelines recommending surgical interventions.

**Case presentation:**

We describe a case of *C. krusei* spondylitis in a patient treated with chemotherapy for acute myeloid leukemia. After induction chemotherapy, the patient developed a candidemia, which was treated with micafungin. One month after the candidemia, the patient was admitted with severe lumbar pain. Spondylitis of the L4 and L5 vertebra was diagnosed on MR-imaging, with signs suggesting an atypical infection. The patient was treated with anidulafungin combined with voriconazole. Despite maximal conservative management symptoms gradually worsened eventually requiring surgical intervention.

**Conclusions:**

In contrast to previous case reports, antimycotic treatment alone could be insufficient in treating *C. krusei* spondylitis.

## Background

Candida spondylitis is an uncommon condition and in most cases due to an infection with *Candida albicans* [1, 2]. *Candida krusei* as a causative agent is rare and only two cases have been reported so far, exclusively in immunocompromised patients [3, 4]. Risk factors for an invasive candida infection are an immunocompromised status, enteric surgery and previous candidemia [3–5]. The previously described cases of *C. krusei* spondylitis were treated with combined antimycotic treatment, although surgical intervention is recommended on indication by the Infectious Diseases Society of America (IDSA) guideline [3, 4, 6].

We describe the first case of *C. krusei* spondylitis in a patient with acute myeloid leukemia, treated with combined antimycotic and surgical intervention.

## Case presentation

A 78-year-old man presented at the emergency department with increasingly disabling lower back pain. His past medical history revealed transurethral resection of the prostate and lumbar spinal stenosis. In addition, he was recently diagnosed with acute myeloid leukemia for which he received induction chemotherapy 1 month earlier. During this course, he developed a perforated diverticulitis for which he underwent resection of the sigmoid. Subsequently, he developed a candidemia with *C. krusei* and micafungin treatment was started. Since ophthalmoscopy, echocardiography and ultrasound of the liver did not reveal any signs of septic foci, micafungin was continued until 14 days after the last positive blood culture. After the first cycle of chemotherapy a complete remission was achieved.

**Fig. 1:**
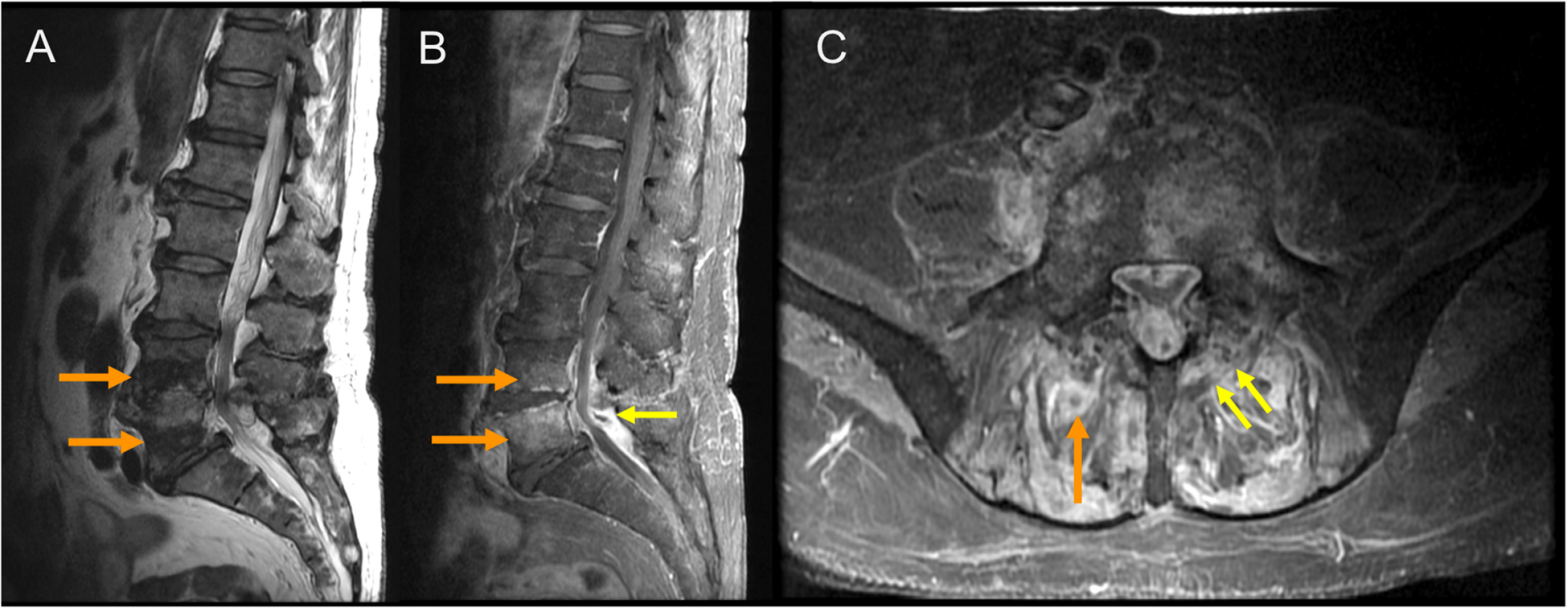
**a** Sagittal T2-weighted turbo spin-echo pulse-sequence shows remarkable low signal intensity in the caudal endplates of L4 and L5 (arrows), as well as the interspinous area. **b** Sagittal T1-weighted spin-echo pulse-sequence with fat sat obtained after administration of intravenous gadolinium reveals pathological contrast enhancement of the caudal endplate of L4 and cranial endplate of L5 (arrows), with sparing of the intervertebral disk L4-L5. Moreover, there is striking enhancement of the intraspinal epidural space at the level of L4-L5 with a small epidural abscess at L5 (small arrow). **c** Axial T1-weighted spin-echo pulse-sequence with fat sat obtained after administration of intravenous gadolinium shows a second small abscess in the right erector spinae muscle (arrow). Note the involvement of the posterior elements, most prominent on the left side (small arrows)

His current complaints started 3 weeks before presentation. He noticed progressive lower back pain radiating to the buttocks and hips hampering his walking. He did not experience loss of motor or sensory function. On presentation, the patient was hemodynamically stable without fever. Neurological and physical examination were normal except for a rigid gait. The patient weight was 77 kg. Blood chemistry showed a slightly elevated CRP of 38 mg/L (normal range < 8 mg/L) and a mild leukocytosis of 12.9 × 10^9^/L (normal range 4–10 × 10^9^/L). An MRI of the lower lumbar spine showed a spondylitis of the L4 and L5 vertebral bodies with remarkable sparing of the intervertebral disc (Fig. 1a). Furthermore, intraspinal epidural expansion of inflammation with an abscess in the posterior epidural space was revealed with notable involvement of the posterior elements (Fig. 1b). A small second abscess in the right erector spinae muscle was punctured and aspirated for culture (Fig. 1c). After puncture, empirical treatment with flucloxacillin (6000 mg/24 h IV) was started. In addition, with his recent candidemia in mind and MRI findings suggesting an atypical pathogen, anidulafungin (loading dose of 200 mg IV, followed by 100 mg/day IV) was added to the treatment. After 6 days culture of the abscess showed *C. krusei*. Susceptibility testing showed the following minimal inhibitory concentrations (MIC): for amphotericin B MIC 0.5 mg/ml, anidulafungin 0.023 mg/L and voriconazole 0.5 mg/mL. Antimicrobial therapy was narrowed to anidulafungin. Because of the low bone penetration of anidulafungin, oral voriconazole (200 mg/twice daily) was added. Considering the infection and risk of derailment upon chemotherapy induced neutropenia, further treatment for acute myeloid leukemia was halted.

Three months after starting the anidulafungin plus voriconazole regimen with adequately controlled serum levels, a gradual increase in backpain and expansion of inflammation on MR-imaging warranted posterior decompression and stabilization from the L2 vertebra to the pelvis. Voriconazole was switched to liposomal amphotericin B (3 mg/kg). Culture of a peroperative bone biopsy (level L4–5) showed persistent infection with *C. krusei*, sensitive to anidulafungin (MIC 0.016 mg/L) and with a low MIC for voriconazol (MIC ≤0.12 mg/L, no clinical breakpoint for *C. krusei*). In this material, no anidulafungin could be detected by high-performance liquid chromatography, suggesting insufficient penetration in bone. Concentrations of voriconazol were not measured due to limited available biopsy material. Determing levels of liposomal amphoterocin B seemed redundant, as this had not been started at the time of biopsy. Nevertheless, due to severe muscle cramps, amphotericin B therapy was switched back to voriconazole, with a trough concentration of 3 mg/l, and the daily dose of anidulafungin was increased to 300 mg. As serum concentration of anidulafungin did not correlate to bone concentration there was no target concentration. Serum levels were monitored through concentrations. At a anidulafungin dose of 300 mg/day the trough concentrations were between 9.9 and 18.7 mg/L. This in contrast to through concentrations of 2.8 to 3.5 mg/L when anidulafungin was dosed 100 mg/day. This treatment will be continued until 6 months after surgery. Complete relieve of symptoms was noted after surgery and the patient experiences no side effects of the current treatment. Unfortunately, 7 months after initial chemotherapeutic treatment his acute myeloid leukemia relapsed. He currently undergoes treatment with azacitidine.

## Discussion and conclusions

Osteomyelitis - including spondylitis - due to Candida species is rare. A recent retrospective study in a tertiary hospital in the United States counted only 15 cases of Candida vertebral osteomyelitis in 12 years [1]. Risk factors are a previous candidemia, prior use of antibiotics, previous (enteric) surgery, central venous catheters and diabetes mellitus [3, 5, 7]. Osteomyelitis is most commonly caused by hematogenous spread of the pathogen, for instance after infection of the urogenitary tract or skin. In our patient, his diverticular disease probably contributed to the infection via the mesenterial veins who have a direct relationship with the paravertebral plexus and the basic vertebral vein of a vertebra. Most cases are due to *C. albicans* [2]. To date, only two cases of spondylitis caused by *C. krusei* have been described, both in patients with acute myeloid leukemia (Table 1) [3, 4].

**Table 1:** Previous cases of C. krusei osteomyelitis

Reference	Species	Location	Sex/age	Symptoms and signs	Risk factors	Treatment	Results/follow-up
Kaldau et al., 2011 [8]	*C. krusei* and *C. tropicalis*	Feet	M/60 y	Pain, swell, leukocytoclastic vasculitis; BC positive	AB	IV Fluconazole and voriconazole 7 days, switch to AmB for 4 mo, relapse, multiple surgeries eventually amputation.	Resolved after relapse /12 months
Peman et al., 2006 [3]	*C. Krusei*	Vertebra	M/62 y	Fever, myalgia, painful skin nodules; BC positive	AML, chemotherapy, neutropenia	IV AmB 2 weeks + caspofungin 4 wks followed by itraconazole PO 4 weeks. After relapse caspofungin + voriconazol 6 wks followed by voriconazol PO	Resolved after relapse/6 months
Schilling et al., 2008 [4]	*C. Krusei*	Vertebra	M/58 y	Lumbar pain, fever; BC positive	AML, chemotherapy, neutropenia, AB	Voriconazole IV 9 days, switch to caspofungin due to ongoing fever for 14 days. Fosofomycin and rifampicin. After 4 mo surgery was performed, caspofungin reinitiated, after 1 mo posaconazole was added for a total of 1 y	Resolved/ 24 months

*AB* antibiotics, *AmB* liposomal amphotericin B, *AML* acute myeloid leukemia, *BC* blood culture, *IV* intravenous, *M* male, *mo* months, *PO* per os, *y* years

On MR imaging, an atypical spondylitis with a yeast should be considered in case of lesions involving contiguous vertebrae without intervertebral disk destruction, a paraspinal inflammatory mass of unusually low signal intensity on T2-sequence, and the presence of multiple small paraspinal abscesses. It is important to take this into account to better guide antibiotic treatment [9, 10].

There are differences in the susceptibility of *C. krusei* isolates for voriconazole across the globe, with lowest sensitivity rates observed in Latin America (74.8%). In Western countries *C. krusei* is usually susceptible to voriconazole. Furthermore, susceptibility for echinocandins is generally good [3]. The Infectious Diseases Society of America (IDSA) guideline on management of candidiasis recommends the use of fluconazole for 6–12 months or an echinocandin for at least 2 weeks followed by fluconazole for 6–12 months in patients with a candida osteomyelitis. Although evidence on combination therapy with echinocandins and azoles is lacking, a combination of voriconazole and an echinocandin is more sensible in case of a *C. krusei* osteomyelitis. In the other two cases of *C. krusei* spondylitis, monotherapy with either amphotericine B, itraconazole, voriconazole, or caspofungin failed. However, combinations of caspofungin with posaconazol [4] or voriconazole [3] were successfully used. There are no known interactions between voriconazole and echinocandins. Surgical debridement is recommended on indication in patients with neurological deficits, spinal instability, large abscesses, or persistent or worsening symptoms or inadequate source control during therapy [6]. In our patient the symptoms worsened despite adequate combined therapy of voriconazole and anidulafungin for 5 months and *C. krusei* could still be cultured from the lesion. Surgery was required for source control and stabilization of the lumbar spine.

Although *C. krusei* spondylitis is a rare condition, a history of backpain in an immunocompromised patient with a history of candidemia should warrant MR-imaging. In addition, despite long-term treatment of a *C. krusei* spondylitis with adequate serum levels of antimycotic therapy, our case illustrates that source control through surgical debridement may be inevitable.

## Data Availability

Data sharing is not applicable to this article as no datasets were generated or analysed during the current study.

## Abbreviations

*C. albicans*: *Candida albicans*
*C. krusei*: *Candida krusei*
CRP: C-reactive protein
H: Hour
IDSA: Infectious diseases society of America
Iv: Intravenous
MIC: Minimal inhibitory concentrations
Mg: Milligram
Ml: Milliliter
MRI: Magnetic resonance imaging
